# Hemoglobin A1c Reduction With the GLP-1 Receptor Agonist Semaglutide Is Independent of Baseline eGFR: *post hoc* Analysis of the SUSTAIN and PIONEER Programs

**DOI:** 10.1016/j.ekir.2022.07.167

**Published:** 2022-08-03

**Authors:** David Z.I. Cherney, Samy Hadjadj, Jack Lawson, Ofri Mosenzon, Katherine Tuttle, Blaz Vrhnjak, Søren Rasmussen, Stephen C. Bain

**Affiliations:** 1 Division of Nephrology, University Health Network, University of Toronto, Toronto, Ontario, Canada; 2 L’institut du thorax, Université de Nantes, CHU Nantes, Centre National de le Recherche Scientifique, Inserm, Nantes, France; 3 Novo Nordisk, Søborg, Denmark; 4 The Diabetes Unit, Department of Endocrinology and Metabolism, Hadassah Medical Center, The Faculty of Medicine, Hebrew University of Jerusalem, Jerusalem, Israel; 5 Providence Health Care, University of Washington, Washington, USA; 6 Swansea University Medical School, Swansea, Wales, UK

**Keywords:** chronic kidney disease, diabetes

## Abstract

**Introduction:**

Glucagon-like peptide-1 receptor agonists (GLP-1RAs) are effective treatments for reducing hemoglobin A1c (HbA1c) in people with type 2 diabetes (T2D), including those with reduced kidney function.

**Methods:**

This *post hoc* analysis assessed the HbA1c-lowering efficacy of semaglutide, a GLP-1RA, in participants with a range of kidney functions in the SUSTAIN 4–6 and 10 (subcutaneous semaglutide) and PIONEER 5 and 6 (oral semaglutide) clinical trials. Trial-level changes from baseline to end of treatment (EOT) in HbA1c and body weight (BW) were assessed in participants with estimated glomerular filtration rate (eGFR) >15 ml/min per 1.73 m^2^ by subgroups categorized according to baseline eGFR. Adverse events were also evaluated.

**Results:**

The analysis included 8859 participants. The mean comparator-adjusted reduction in HbA1c from baseline to EOT with semaglutide ranged from 0.6% to 1.6% points across trials, with similar reductions across the eGFR subgroups (interaction *P*-value ≥ 0.33 for difference between eGFR subgroups within each trial). Greater weight loss from baseline to EOT with semaglutide versus comparator was observed across almost all baseline eGFR subgroups, with nominally greater weight loss with lower versus higher eGFR in SUSTAIN 6 and 10 and PIONEER 5 and 6 (interaction *P* < 0.05). No new safety concerns with semaglutide were identified.

**Conclusion:**

The HbA1c-lowering effect of semaglutide in participants with T2D was comparable irrespective of eGFR, which ranged upwards from eGFR >15 ml/min per 1.73 m^2^.

## Graphical abstract

**Figure:**
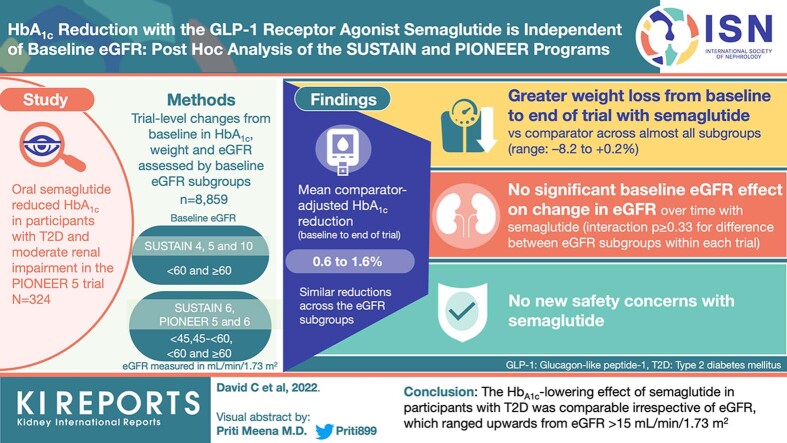

See Commentary on Page 2323

The cornerstone of T2D management is glycemic control.^1^ Nevertheless, not all glucose-lowering agents are suitable for all people with T2D and reduced kidney function. For example, metformin should not be introduced or, if metformin is already used, the dose should be reviewed in patients with an eGFR <45 ml/min per 1.73 m^2^ and it is contraindicated for those with an eGFR of <30 ml/min per 1.73 m^2^. Sulfonylureas are associated with greater risk for hypoglycemic events in patients with reduced eGFR compared with those without reduced kidney function.^2^ Sodium–glucose cotransporter-2 inhibitors have reduced glycemic-lowering efficacy in people with eGFR <60 ml/min per 1.73 m^2^ and are not recommended for use in patients with eGFR <30 ml/min per 1.73 m^2^.^3^, ^4^, ^5^, ^6^

Semaglutide, a GLP-1RA, is approved in several countries for the treatment of T2D in 2 different formulations as follows: (i) subcutaneous (s.c.) once-weekly (OW) and (ii) oral once-daily (OD).^7^^,^^8^ In the phase 3 SUSTAIN and PIONEER clinical trial programs, OW s.c. semaglutide and OD oral semaglutide, respectively, consistently demonstrated superior, clinically relevant reductions in HbA1c and BW compared with placebo and active comparators in adults with T2D; the safety profile of semaglutide was consistent with its class.^9^, ^10^, ^11^, ^12^, ^13^, ^14^, ^15^, ^16^, ^17^, ^18^, ^19^, ^20^ There is no apparent effect of reduced kidney function or hemodialysis on the pharmacokinetics of semaglutide,^21^ and no dose adjustment of semaglutide (s.c. or oral) is required in patients with reduced kidney function.^7^^,^^8^ These findings are supported by the placebo-controlled, phase 3a PIONEER 5 trial, in which oral semaglutide was shown to be effective in patients with T2D and moderate renal impairment,^22^ as well as by studies of other GLP-1RAs, such as dulaglutide.^23^

The aim of this *post hoc* analysis was to assess the glycemic-lowering efficacy of semaglutide in participants across a range of eGFR levels in the SUSTAIN (s.c. semaglutide) and PIONEER (oral semaglutide) clinical trial programs.

## Methods

This *post hoc*, trial-level analysis considered trials from the SUSTAIN and PIONEER programs that had enrolled >10 participants with eGFR <60 ml/min per 1.73 m^2^. Six trials met these criteria: SUSTAIN 4, 5, 6, and 10, and PIONEER 5 and 6. Data were evaluated for each trial separately and *post hoc* exploratory analyses were performed to compare outcomes for all participants (full analysis set) stratified by baseline eGFR.

### Design of the SUSTAIN and PIONEER Trials

The trial designs of SUSTAIN 4–6 and 10, and PIONEER 5 and 6 have been reported previously and are summarized in Supplementary Table S1; all trials were registered with ClinicalTrials.gov (NCT02128932, NCT02305381, NCT01720446, NCT03191396, NCT02827708, and NCT02692716). The SUSTAIN trials investigated s.c. OW semaglutide up to a maximum dose of 1.0 mg, whereas the PIONEER trials investigated oral OD semaglutide up to a maximum dose of 14 mg.

### Participants

The inclusion and exclusion criteria of the trials were broadly similar. Participants were adults (age ≥18 years) with T2D and HbA1c 7.0% to 10.0% (SUSTAIN 4 and 5), HbA1c ≥7% (SUSTAIN 6), HbA1c 7.0% to 11.0% (SUSTAIN 10), and HbA1c 7.0% to 9.5% (PIONEER 5). In PIONEER 6, HbA1c was not a criterion for inclusion or exclusion. In the SUSTAIN 6 and PIONEER 6 cardiovascular (CV) outcomes trials, eligible participants were aged ≥50 years old with established CV disease or chronic kidney disease (CKD), or ≥60 years old with CV risk factors only. Serum creatinine was assessed at week –2 for SUSTAIN 4 and 5, and 10, and at baseline (week 0) for SUSTAIN 6 and PIONEER 5 and 6, and thereafter at regular intervals throughout the treatment periods for all trials.

All trials were conducted in compliance with the Declaration of Helsinki^24^ and the Guidelines for Good Pharmacoepidemiology Practices. The protocols were approved by Independent Local Ethics Committees and Institutional Review Boards at each participating center. Participants provided informed consent before the commencement of any study-related activities.

### eGFR Subgroups

Subgroup cut-offs for baseline eGFR analyses were based on clinical cut-offs recommended by the Kidney Disease: Improving Global Outcomes guidelines for CKD staging.^6^ The eGFR cut-offs selected (in ml/min per 1.73 m^2^) were: <60 and ≥60 for SUSTAIN 4, 5, and 10 (in which the enrolled study populations did not include enough renal-impaired participants to statistically power a lower cutoff group) and <45, 45 to <60, and ≥60 for SUSTAIN 6 and PIONEER 5 and 6 (in which the study populations included participants with moderate kidney impairment).

### Outcomes

Placebo- and active-comparator-adjusted change from baseline to EOT by baseline eGFR subgroup was assessed *post hoc* within each trial for the following: HbA1c (% points) and BW (% [confirmatory end points]), systolic BP and diastolic BP (mmHg). Safety assessments included the incidence of adverse events (AEs; including gastrointestinal [GI] and severe hypoglycemic episodes).

### Statistical Analysis

The following parameters were analyzed from baseline: relative change in HbA1c (% points), change in BW (%), and change in BP (mmHg). Within each trial, a linear mixed model with repeated measures across visits was used to compare absolute estimated change in the relevant parameter from baseline to EOT between eGFR subgroups. Data from participants who were on randomized treatment and without rescue medication or prematurely discontinued were included in the analyses, except for SUSTAIN 6 and PIONEER 6, in which all in-trial data for randomized participants were included. The model used allocated treatment, eGFR subgroup, and treatment-by-eGFR subgroup interaction as fixed effects and relevant baseline values as covariates, including HbA1c, all nested within visits, and an unstructured residual covariance matrix. Change from baseline in HbA1c (% points) at EOT with baseline eGFR as a continuous variable was also analyzed using a mixed model with repeated measures quadratic spline function.

Data from all trials were analyzed separately. Data for participants receiving semaglutide 0.5 mg and 1.0 mg in SUSTAIN 4, 5, and 6 were pooled within each individual trial in the analyses, where relevant. The interaction *P*-value for treatment-by-eGFR was evaluated at EOT. *P* < 0.05 was considered statistically significant. No adjustment for multiplicity was performed.

### Role of the Funding Source

The sponsor, Novo Nordisk, designed the clinical trials and was responsible for site monitoring, data collection, data analysis, and data interpretation. The sponsor also funded editorial support, provided by independent medical writers. All authors participated in designing the *post hoc* analyses, planning and review of the manuscript and had full access to all the data in the studies on request. Author, Søren Rasmussen (Novo Nordisk) takes responsibility for the integrity and accuracy of the data analysis. The authors made the final decision to submit for publication.

## Results

### Disposition and Baseline Characteristics

Across the SUSTAIN 4–6 and 10, and PIONEER 5 and 6 trials, adults with T2D were randomly assigned to receive OW semaglutide, OD semaglutide 14 mg, active comparator, or placebo (Supplementary Table S1). A total of 8859 participants were included in the analyses from the 6 trials investigating OW or OD semaglutide versus comparators (Table 1); SUSTAIN 4 (OW semaglutide vs. OD insulin glargine); SUSTAIN 5 (OW semaglutide vs. placebo); SUSTAIN 6 (OW semaglutide vs. placebo); SUSTAIN 10 (OW semaglutide vs. OD liraglutide); PIONEER 5 (OD semaglutide vs. placebo); and PIONEER 6 (OD semaglutide vs. placebo). The baseline characteristics of participants are summarized by trial and baseline eGFR in Supplementary Table S2. Mean baseline HbA1c ranged from 8.0% to 8.7%, and mean baseline BW ranged from 90.8 kg to 96.9 kg. Larger proportions of participants in SUSTAIN 6 (25.2%), PIONEER 5 (90.5%), and PIONEER 6 (26.9%) had moderate-to-severe kidney impairment (i.e., eGFR ≥15–<60 ml/min per 1.73 m^2^) than in SUSTAIN 4 (4.0%), SUSTAIN 5 (7.3%), and SUSTAIN 10 (5.0%). Across trials, participants with a lower baseline eGFR were generally older and less likely to be receiving metformin at baseline than those with a higher baseline eGFR.

**Table 1:** Participant disposition and baseline characteristics by trial

Characteristics	SUSTAIN 4 30 wk	SUSTAIN 5 30 wk	SUSTAIN 6 104 wk	SUSTAIN 10 30 wk	PIONEER 5 26 wk	PIONEER 6 ≤83 wk
Participant disposition, n (%)						
Randomized^a^	1082	396	3297	577	324	3183
Trial completers	1020	380	3232	569	314	3172
Discontinued treatment	130 (12.0)	43 (10.9)	660 (20.0)	66 (11.4)	41 (12.7)	400 (12.6)
Baseline characteristics, mean (SD)						
Age, mean (SD), years	56.5 (10.4)	58.8 (10.1)	64.6 (7.4)	59.5 (10.2)	70.4 (7.9)	66.1 (7.1)
Female, n (%)	508 (47.0)	174 (43.9)	1295 (39.3)	250 (43.3)	168 (51.9)	1007 (31.6)
HbA1c, mean (SD), %	8.2 (0.9)	8.4 (0.8)	8.7 (1.5)	8.2 (1.0)	8.0 (0.7)	8.2 (1.6)
Body weight, mean (SD), kg	93.4 (21.8)	91.7 (21.0)	92.1 (20.6)	96.9 (21.3)	90.8 (17.6)	90.9 (21.2)
Diabetes duration, mean (SD), year	8.6 (6.3)	13.3 (7.8)	13.9 (8.1)	9.3 (5.9)	14.0 (8.0)	14.9 (8.5)
eGFR (CKD-EPI), mean (SD)	96.1 (17.8)	90.3 (18.6)	75.7 (22.9)	92.4 (17.3)	47.6 (9.7)	74.2 (21.0)
Kidney impairment, n (%)						
None (eGFR ≥90)	751 (69.4)	229 (57.8)	1119 (33.9)	369 (64.0)	0.0	919 (28.9)
Mild (eGFR ≥60 to <90)	288 (26.6)	138 (34.8)	1308 (39.7)	179 (31.0)	31 (9.6)	1389 (43.6)
Moderate (eGFR ≥30 to <60)	43 (4.0)	29 (7.3)	733 (22.2)	29 (5.0)	285 (88.0)	827 (26.0)
Severe (eGFR ≥15 to <30)	0.0	0.0	100 (3.0)	0.0	8 (2.5)	28 (0.9)
Metformin use at baseline, n (%)	1082 (100)	330 (83.3)	2414 (73.3)	547 (94.8)	242 (74.7)	2464 (77.4)
SBP, mmHg, mean (SD)	132.1 (15.3)	134.8 (16.0)	135.6 (17.1)	136.4 (14.8)	137.5 (15.1)	135.6 (17.6)
DBP, mmHg, mean (SD)	79.9 (8.5)	79.0 (9.8)	70.0 (10.0)	81.2 (9.4)	77.6 (9.1)	76.0 (10.1)
UACR, mg/g, geometric mean (% covariance)	14.7 (257.2)	23.1 (373.3)	24.2 (743.8)	Not available	Not available	Not available

CKD-EPI, Chronic Kidney Disease Epidemiology Collaboration; DBP, diastolic blood pressure; eGFR, estimated glomerular filtration rate (measured in ml/min per 1.73 m^2^); HbA1c, hemoglobin A1c; SBP, systolic blood pressure; UACR, urinary albumin–creatinine ratio.

a Randomized with a valid eGFR value at baseline.

### HbA1c

**Figure 1:**
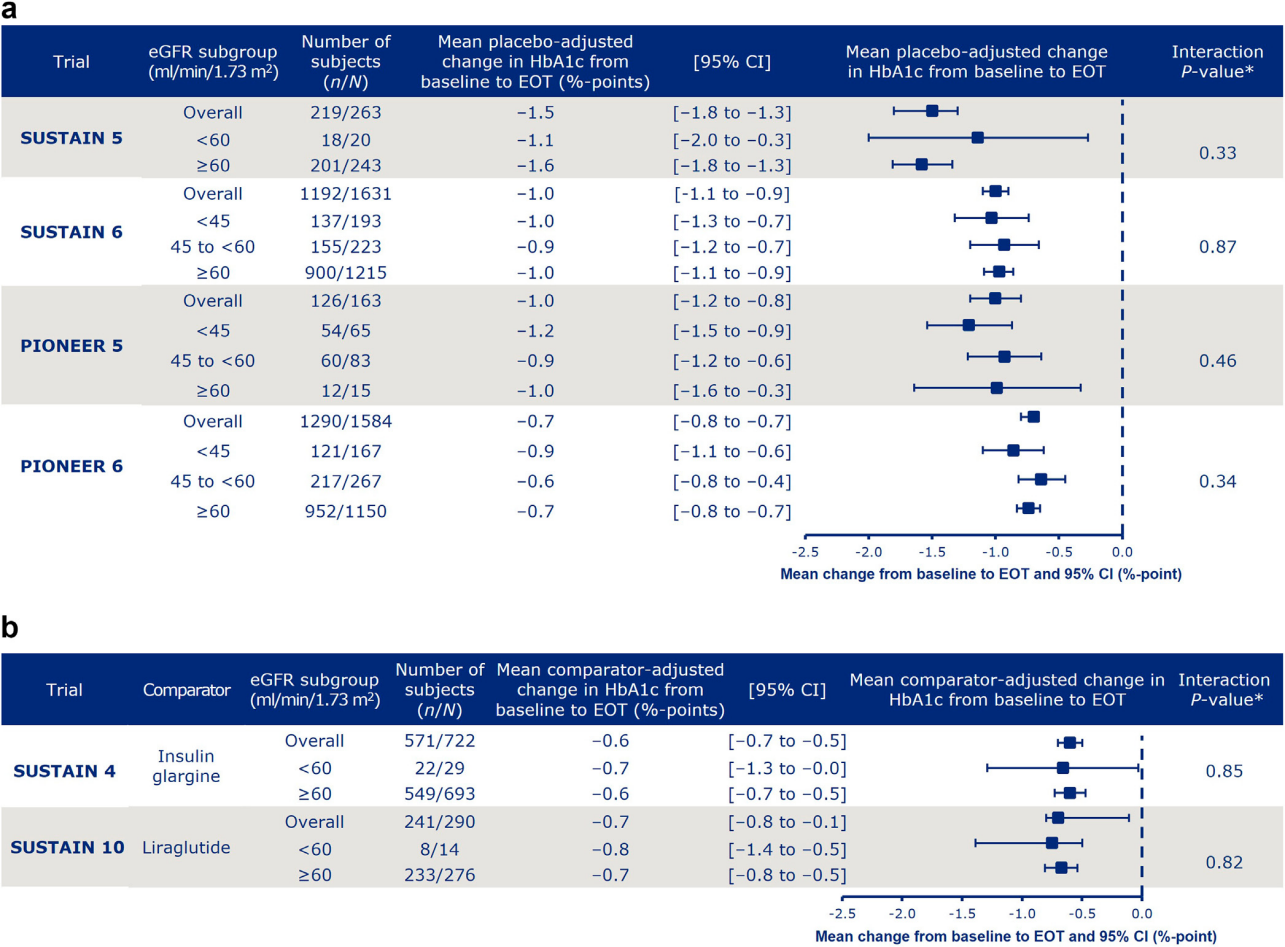
Mean placebo (a) and active-comparator-adjusted (b) change in HbA1c from baseline to EOT with semaglutide, by eGFR subgroup. ∗Interaction between treatment and HbA1c at EOT. Data are from the full analysis set. Data from participants who were on randomized treatment and without rescue medication or prematurely discontinued were included in the analyses, except for SUSTAIN 6 and PIONEER 6, for which all in-trial data for randomized participants were included. eGFR, estimated glomerular filtration rate; EOT, end of treatment; HbA1c, hemoglobulin A1c; N, number of participants in the full analysis set who received semaglutide; n, number of participants who received semaglutide and contributed to the analysis.

The mean placebo- and active comparator-adjusted reduction in HbA1c from baseline to EOT with semaglutide ranged from 0.6% points to 1.6% points across trials and eGFR subgroups and, within each trial, placebo- and active comparator-adjusted mean reductions in HbA1c were similar across the eGFR subgroups (interaction *P* ≥ 0.33 for difference between eGFR subgroups within each trial) (Figure 1a and b). When treatment difference was analyzed with eGFR as a continuous variable, the results were broadly consistent across trials regardless of baseline eGFR (Supplementary Figure S1). Reductions in HbA1c from baseline to EOT within each trial were also similar across the eGFR subgroups in participants in the semaglutide and comparator arms (placebo, liraglutide, and insulin glargine) and ranged from 1.0% points to 1.7% points in participants treated with semaglutide (Supplementary Figure S2).

### Body Weight Changes

**Figure 2:**
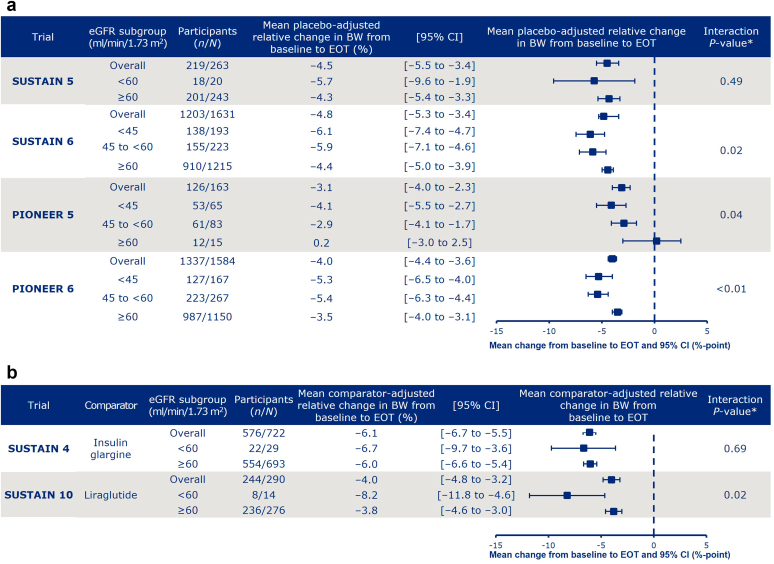
Relative change from baseline to EOT in BW (%) in placebo (a) and active comparator (b) trials. ∗Interaction between treatment and BW at EOT. Data are from the full analysis set. Data from participants who were on randomized treatment and without rescue medication or prematurely discontinued were included in the analyses, except for SUSTAIN 6 and PIONEER 6, for which all in-trial data for randomized participants were included. BW, body weight; eGFR, estimated glomerular filtration rate; EOT, end of treatment; N, number of participants in the full analysis set who received semaglutide; n, number of participants who received semaglutide and contributed to the analysis.

The mean placebo- and active comparator-adjusted relative change in BW from baseline to EOT with semaglutide ranged from –8.2% to +0.2% across trials and eGFR subgroups (Figure 2a and b). Reductions in BW were observed with semaglutide treatment in all the eGFR subgroups except for the ≥60 ml/min per 1.73 m^2^ subgroup in PIONEER 5. The treatment-by-subgroup interaction test indicated statistically significant differences between the baseline eGFR subgroups in SUSTAIN 6 and 10 and PIONEER 5 and 6 (interaction *P* < 0.05), such that weight loss was nominally greater in the subgroup with a lower eGFR.

### Blood Pressure Changes

**Figure 3:**
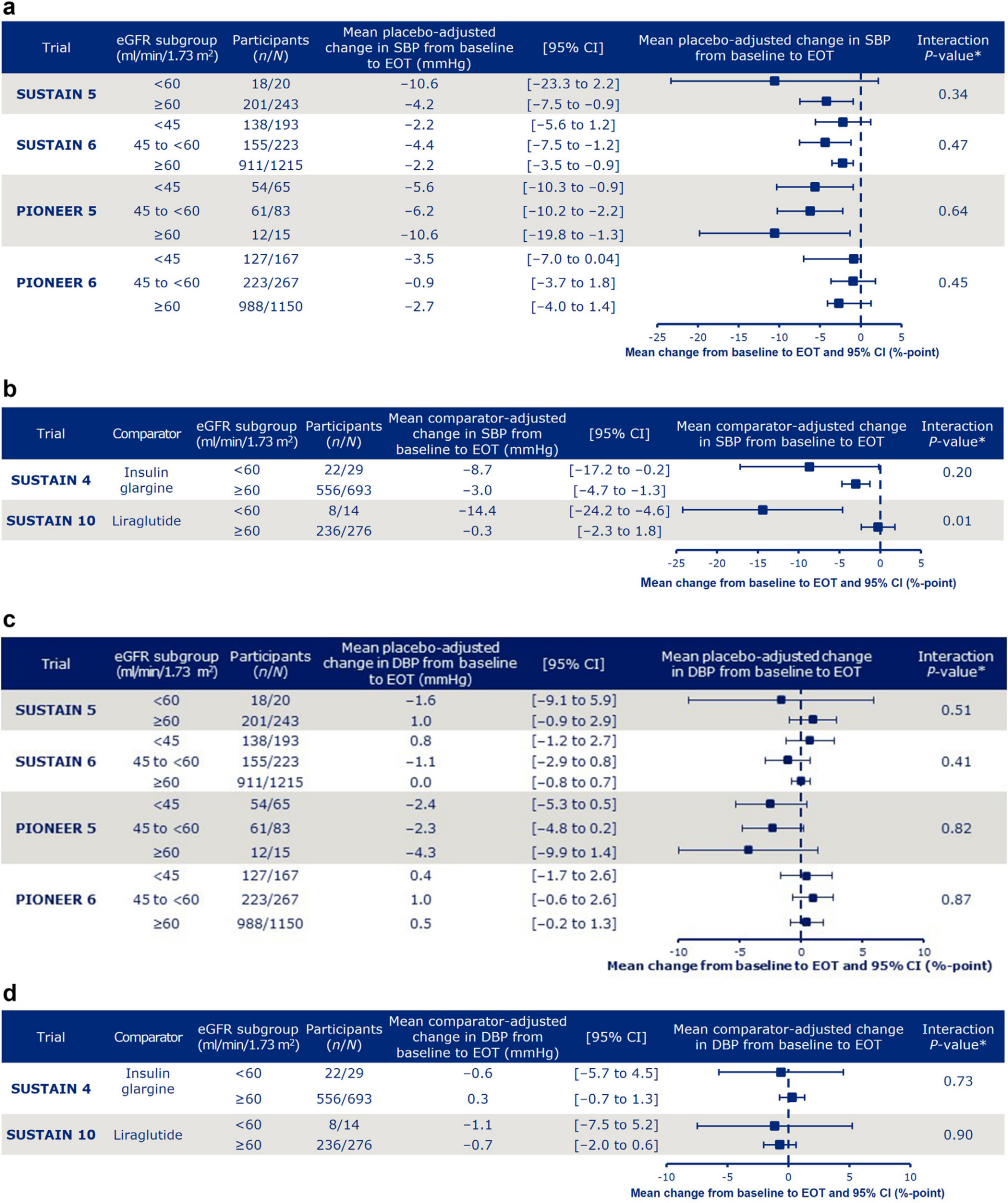
Change from baseline to EOT in (a, b) systolic and (c, d) diastolic blood pressure parameters in placebo (a, c) and active comparator (b, d) trials. ∗Interaction between treatment and eGFR at EOT. Data are from the full analysis set. Data from participants who were on randomized treatment and without rescue medication or prematurely discontinued were included in the analyses, except for SUSTAIN 6 and PIONEER 6, for which all in-trial data for randomized participants were included. DBP, diastolic blood pressure; eGFR, estimated glomerular filtration rate; EOT, end of treatment; N, number of participants in the full analysis set who received semaglutide; n, number of participants who received semaglutide and contributed to the analysis; SBP, systolic blood pressure.

Across trials and eGFR subgroups, the mean placebo- and active comparator-adjusted change from baseline to EOT with semaglutide ranged from –14.4 to –0.3 mmHg for systolic BP and from –4.3 to 1.0 mmHg for diastolic BP (Figure 3a-d). Across trials, there was generally a homogeneous effect of baseline eGFR level on treatment differences in change of BP from baseline, as indicated by the interaction test between treatment and eGFR subgroups, with the exception of systolic BP in SUSTAIN 10 (interaction *P*-value = 0.01).

### Safety

A summary of AEs in SUSTAIN 4–6 and 10, and PIONEER 5 and 6 is shown in Tables 2 to 4. Generally, a higher proportion of participants had serious AEs in the patients with eGFR <60 ml/min/1.73 m^2^ versus those with eGFR ≥60 ml/min/1.73 m^2^ across treatment arms (Supplementary Table S3). The proportion of participants treated with semaglutide who reported GI AEs ranged from 16.2% to 55.6%, with comparable rates across eGFR subgroups. Severe hypoglycemic episodes were uncommon. Numerically, eGFR <60 ml/min/1.73 m^2^, was associated with a greater risk for hypoglycemia compared with eGFR ≥60 ml/min/1.73 m^2^ across all treatment arms. Fatal events were clustered in the 2 CV outcomes trials, SUSTAIN 6 and PIONEER 6. In SUSTAIN 6, the proportions of deaths were higher in the 2 subgroups with eGFR <60 ml/min per 1.73 m^2^ compared with the subgroup with eGFR ≥60 ml/min per 1.73 m^2^ in both the semaglutide and the placebo arms.

**Table 2:** Summary of adverse events in SUSTAIN 4 and 5^a^

Study	SUSTAIN 4				SUSTAIN 5			
Baseline eGFR (CKD-EPI)	<60		≥60		<60		≥60	
	Semaglutide (*n* = 29)	Insulin glargine (*n* = 14)	Semaglutide (*n* = 693)	Insulin glargine (*n* = 346)	Semaglutide (*n* = 20)	Placebo (*n* = 9)	Semaglutide (*n* = 243)	Placebo (*n* = 124)
AEs	23 (79.3)	10 (71.4)	497 (71.7)	231 (66.8)	18 (90.0)	5 (55.6)	157 (64.6)	74 (59.7)
Serious AEs	4 (13.8)	0 (0)	36 (5.2)	18 (5.2)	4 (20.0)	1 (11.1)	16 (6.6)	8 (6.5)
Severe AEs	3 (10.3)	0 (0)	46 (6.6)	10 (2.9)	3 (15.0)	1 (11.1)	12 (4.9)	5 (4.0)
Fatal AEs	0 (0)	0 (0)	4 (0.6)	2 (0.6)	0 (0)	0 (0)	0 (0)	0 (0)
GI AEs	14 (48.3)	2 (14.3)	291 (42.0)	52 (15.0)	8 (40.0)	3 (33.3)	73 (30.0)	18 (14.5)
Severe hypoglycemic episodes (ADA)	0 (0)	0 (0)	7 (1.0)	5 (1.4)	1 (5.0)	1 (11.1)	2 (0.8)	0 (0)
Acute kidney failure	0 (0)	0 (0)	4 (0.6)	0 (0)	1 (5.0)	1 (11.1)	1 (0.4)	1 (0.8)
AE leading to premature treatment discontinuation	3 (10.3)	0 (0)	43 (6.2)	5 (1.4)	1 (5.0)	0 (0)	14 (5.8)	1 (0.8)

Data are *n* (%) and are from the full analysis set.

ADA, American Diabetes Association; AE, adverse event; CKD-EPI, Chronic Kidney Disease Epidemiology Collaboration; eGFR, estimated glomerular filtration rate (measured in ml/min per 1.73 m^2^); GI, gastrointestinal.

a AEs are included for participants with ≥1 event.

**Table 3:** Summary of adverse events in SUSTAIN 6 and 10^a^

Study	SUSTAIN 6				SUSTAIN 10			
Baseline eGFR (CKD-EPI)	<60		≥60		<60		≥60	
	Semaglutide (*n* = 416)	Placebo (*n* = 427)	Semaglutide (*n* = 1215)	Placebo (*n* = 1212)	Semaglutide (*n* = 14)	Liraglutide (*n* = 15)	Semaglutide (*n* = 276)	Liraglutide (*n* = 272)
AEs	380 (91.3)	394 (92.3)	1076 (88.6)	1081 (89.2)	13 (92.9)	13 (86.7)	192 (69.6)	177 (65.1)
Serious AEs	182 (43.8)	194 (45.4)	378 (31.1)	429 (35.4)	0 (0)	1 (6.7)	18 (6.5)	22 (8.1)
Severe AEs	138 (33.2)	148 (34.7)	265 (21.8)	258 (21.3)	2 (14.3)	2 (13.3)	16 (5.8)	15 (5.5)
Fatal AEs	23 (5.5)	22 (5.2)	39 (3.2)	38 (3.1)	0 (0)	0 (0)	0 (0)	0 (0)
GI AEs	229 (55.0)	165 (38.6)	612 (50.4)	414 (34.2)	7 (50.0)	5 (33.3)	120 (43.5)	105 (38.6)
Severe hypoglycemic episodes (ADA)	11 (2.6)	18 (4.2)	14 (1.2)	11 (0.9)	0 (0)	0 (0)	0 (0)	0 (0)
Acute kidney failure	39 (9.4)	39 (9.1)	25 (2.1)	28 (2.3)	0 (0)	1 (6.7)	0 (0)	0 (0)
AE leading to premature treatment discontinuation	71 (17.1)	37 (8.7)	143 (11.8)	73 (6.0)	4 (28.6)	1 (6.7)	29 (10.5)	19 (7.0)

Data are *n* (%) and are from the full analysis set.

ADA, American Diabetes Association; AE, adverse event; CKD-EPI, Chronic Kidney Disease Epidemiology Collaboration; eGFR, estimated glomerular filtration rate (measured in ml/min per 1.73 m^2^); GI, gastrointestinal.

a AEs are included for participants with ≥1 event.

**Table 4:** Summary of adverse events in PIONEER 5 and 6^a^

Study	PIONEER 5				PIONEER 6			
Baseline eGFR (CKD-EPI)	<60		≥60		<60		≥60	
	Semaglutide (*n* = 148)	Placebo (n *=* 145)	Semaglutide (*n* = 15)	Placebo (*n* = 16)	Semaglutide (*n* = 434)	Placebo (*n* = 422)	Semaglutide (*n* = 1150)	Placebo (*n* = 1158)
AEs	113 (76.4)	98 (67.6)	9 (60.0)	11 (68.8)	202 (46.5)	158 (37.4)	453 (39.4)	370 (32.0)
Serious AEs	19 (12.8)	16 (11.0)	1 (6.7)	2 (12.5)	117 (27.0)	120 (28.4)	207 (18.0)	249 (21.5)
Severe AEs	9 (6.1)	13 (9.0)	1 (6.7)	2 (12.5)	81 (18.7)	86 (20.4)	147 (12.8)	130 (11.2)
Fatal AEs	0 (0)	1 (0.7)	1 (6.7)	1 (6.3)	12 (2.8)	23 (5.5)	13 (1.1)	23 (2.0)
GI AEs	69 (46.6)	24 (16.6)	5 (33.3)	4 (25.0)	81 (18.7)	21 (5.0)	186 (16.2)	52 (4.5)
Severe hypoglycemic episodes (ADA)	0 (0)	0 (0)	0 (0)	0 (0)	13 (3.0)	3 (0.7)	13 (1.1)	13 (1.1)
Acute kidney failure	4 (2.7)	3 (2.1)	0 (0)	0 (0)	13 (3.0)	12 (2.8)	10 (0.9)	10 (0.9)
AE leading to premature treatment discontinuation	22 (14.9)	8 (5.5)	2 (13.3)	0 (0)	139 (32.0)	89 (21.1)	285 (24.8)	178 (15.4)

Data are *n* (%) and are from the full analysis set.

ADA, American Diabetes Association; AE, adverse event; CKD-EPI, Chronic Kidney Disease Epidemiology Collaboration; eGFR, estimated glomerular filtration rate (measured in ml/min per 1.73 m^2^); GI, gastrointestinal.

a AEs are included for participants with ≥1 event.

## Discussion

This *post hoc* trial-level analysis of the trials from the SUSTAIN and PIONEER programs showed the HbA1c-lowering effect of semaglutide appears to be consistent across different baseline eGFR subgroups in participants with T2D. Participant baseline characteristics were similar for all the eGFR subgroups, except for small differences between HbA1c and BW, and participants with a lower eGFR tended to be older with a longer duration of diabetes than those with a higher eGFR. When analyzed by randomization arm, reductions in HbA1c with semaglutide were superior to those with either active comparator (insulin glargine or liraglutide) or with placebo for all eGFR subgroups, and reductions in BW were also significantly greater with semaglutide versus placebo and active comparators across all but 1 of the subgroups. In some trials there was a trend that reductions in BW (from a mean baseline BW of 90.8–96.9 kg across the SUSTAIN and PIONEER trials included) appeared to be greater in the subgroups with a lower eGFR than in subgroups with a higher eGFR. This indicative finding warrants further investigation. No significant effect of baseline eGFR level on the change from baseline in systolic BP or diastolic BP was observed.

A pooled analysis of results from clinical trials with exenatide extended-release (another OW GLP-1RA), in participants with T2D and stage 2 (mild renal impairment; eGFR ≥60 to <90 ml/min per 1.73 m^2^) or 3 CKD (moderate renal impairment; eGFR ≥30 to <60 ml/min per 1.73 m^2^), showed that changes from baseline to EOT in HbA1c, BW, and systolic BP were similar in all the CKD subgroups receiving exenatide, which is consistent with our findings. AEs leading to treatment discontinuation also appeared more likely with GLP-1RA treatment versus comparators; this effect was not more pronounced in those with renal impairment.^25^

In most of the trials, a higher proportion of participants in the subgroups with lower eGFR experienced AEs than in the subgroups with higher eGFR. Because this finding was consistent with semaglutide, placebo, and the active comparators, this was possibly related to the greater burden of comorbidities in the subgroups with lower eGFR. GI AEs were more common in participants receiving semaglutide than in those receiving placebo or active comparator. These AEs were generally similar across eGFR subgroups, suggesting that baseline eGFR does not greatly affect GI tolerability. These findings were consistent with a single-center, single-dose, parallel-group, open-label trial of patients with varying degrees of renal impairment receiving OD subcutaneous liraglutide, in which GI-related AEs were similar in patients across eGFR subgroups.^26^ Severe hypoglycemic episodes were rare and did not appear to be affected by baseline eGFR. Despite this, the risk of hypoglycemia was more often numerically higher in patients with low eGFR (<60 ml/min per 1.73 m^2^), which is consistent with other published studies.^27^ Although a higher proportion of patients discontinued treatment because of AEs with semaglutide versus comparators, discontinuation because of AEs seemed to be more likely in the subgroups with lower eGFR versus higher eGFR, and this tendency was more pronounced with both semaglutide and comparators.

In addition to its beneficial effects on HbA1c and weight, semaglutide has been shown in CV outcomes trials to have cardio-kidney benefits.^28^^,^^29^ Other GLP-1RAs have also been shown to have cardio-kidney benefits.^29^, ^30^, ^31^, ^32^, ^33^, ^34^, ^35^ In a systematic review and meta-analysis of 7 CV outcomes trials, GLP-1RAs were shown to improve a broad composite kidney disease outcome (development of new-onset macroalbuminuria, decline in eGFR [or increase in creatinine], progression to end-stage kidney disease, or death attributable to kidney causes) by 21%.^32^ In this analysis, the cardioprotective effects on major adverse CV events (a composite of CV death, stroke, or myocardial infarction) were consistent across the CKD subgroups tested, including baseline eGFR <60 ml/min per 1.73 m^2^ versus ≥60 ml/min per 1.73 m^2^. The results from our analysis, and from other studies,^30^, ^31^, ^32^, ^33^, ^34^, ^35^ support the hypothesis that GLP-1RAs may have kidney-protective properties – a concept that is being tested in dedicated clinical trials.

The observations on kidney protection with GLP-1RAs have already been translated to clinical practice guidelines in the American Diabetes Association Standards of Care 2021.^1^ In addition, according to the Kidney Disease: Improving Global Outcomes guidelines, a GLP-1RA is the preferred glucose-lowering agent for patients with diabetes and eGFR <30 ml/min per 1.73 m^2^. The European Society of Cardiology and the European Association for the Study of Diabetes guidelines also recommend that treatment of diabetes with liraglutide, dulaglutide or semaglutide can be considered in patients with eGFR >15 ml/min per 1.73 m^2^. The current report, in demonstrating both a consistent glycemic lowering with semaglutide treatment and a consistent safety profile with the GLP-1RA class in the setting of CKD, is important in the context of the potential greater use of such therapies in nephrology-focused clinical practice.

Our analysis examined multiple trials across the semaglutide phase 3 trial programs, which included participants with a wide range of kidney function. Nevertheless, an important limitation is that the analysis was performed *post hoc*, and some of the subgroups examined contained small numbers of participants, which hinders the interpretation of some results. In addition, the trials included had not been designed to address kidney status, so were not powered to evaluate effects on CKD outcomes. In some of the PIONEER trials, for example, urinary albumin–to–creatinine ratio data were not collected. Furthermore, comparators across trials differed, as did trial length, population, and CV and CKD risk. A further limitation of the analysis is the missing data that resulted from patients discontinuing the trial because of AEs and, in particular, because of fatal AEs. Lastly, there were small differences in baseline HbA1c values between the eGFR subgroups in the trials, which was a limitation because baseline HbA1c may impact the effect of a treatment on HbA1c level.

This *post hoc* analysis demonstrates the antihyperglycemic effect of semaglutide in participants with T2D and reduced kidney function. Beyond this analysis, the potential benefit of semaglutide in delaying the progression of kidney impairment in participants with T2D and CKD is the subject of the ongoing FLOW study (NCT03819153), which has primary kidney disease end points. In addition, the mechanistic REMODEL trial (NCT04865770) aims to assess the potential mode of action of semaglutide using advanced imaging modalities and kidney biopsy studies in subjects with T2D and CKD.

## Disclosure

DZIC has received honoraria from Boehringer Ingelheim-Eli Lilly, Merck, AstraZeneca, Sanofi, Mitsubishi-Tanabe, AbbVie, Janssen, Bayer, Prometic, Bristol-Myers Squibb, Maze, CSL-Behring, and Novo Nordisk, and has received operational funding for clinical trials from Boehringer Ingelheim-Eli Lilly, Merck, Janssen, Sanofi, AstraZeneca, and Novo Nordisk. SH reports personal fees and nonfinancial support from AstraZeneca, grants and personal fees from Bayer, personal fees from Boehringer Ingelheim, grants from Dinno Santé, personal fees from Eli Lilly, nonfinancial support from LVL, personal fees and nonfinancial support from Merck Sharp & Dohme, personal fees from Novartis, grants from Pierre Fabre Santé, personal fees and nonfinancial support from Sanofi, personal fees and nonfinancial support from Servier, personal fees from Valbiotis. JL, BV, and SR are employees of Novo Nordisk A/S. SR also holds stock in Novo Nordisk A/S. OM reports grant for advisory board, speakers’ bureau, through Hadassah University Hospital, medical writing, support for travel, article processing charges from Novo Nordisk, a grant through Hadassah University Hospital from AstraZeneca, speakers’ bureau from Eli Lilly, Sanofi, Merck Sharp & Dohme, Boehringer Ingelheim, Novartis, AstraZeneca, and BOL Pharma, support for travel/meetings from AstraZeneca, and advisory board from Eli Lilly, Sanofi, Merck Sharp & Dohme, Boehringer Ingelheim, and BOL Pharma. KT reports grants/contracts (paid to institution) from the National Institute of Diabetes and Digestive and Kidney Diseases/National Institutes of Health, National Heart, Lung, and Blood Institute/National Institutes of Health, National Center for Advancing Translational Sciences//National Institutes of Health, Centers for Disease Control and Prevention and Travere, consulting fees from Astra Zeneca, Bayer, Boehringer Ingelheim, Eli Lilly, Gilead, Goldfinch Bio, and Novo Nordisk, honoraria from Astra Zeneca, Bayer, Eli Lilly, Gilead, Goldfinch Bio, and support for attending meetings and/or travel from Eli Lilly and Novo Nordisk. She participated on a Data Safety Monitoring Board or Advisory Board for the National Institute of Diabetes and Digestive and Kidney Diseases/National Institutes of Health and George Clinical Institute. She was also the Chair for the Diabetic Kidney Disease Collaborative Task Force, American Society of Nephrology, and the Board of Directors, Kidney Health Initiative, United States Food and Drug Administration and the American Society of Nephrology. SB received support for a medical writing agency, honoraria and support for attending the virtual European Association for the Study of Diabetes meeting in 2021 from Novo Nordisk.

## Supplementary Material

Supplementary File (PDF)

**Table S1.** Trial designs.

**Table S2.** Demographics and baseline characteristics by trial and baseline eGFR in (A) SUSTAIN 4 and 5, (B) SUSTAIN 6 and 10, and (C) PIONEER 5 and 6.

**Table S3.** Summary of adverse events in (A) SUSTAIN 6 and 10, and (B) PIONEER 5 and 6 by baseline eGFR <45, 45 to <60 and ≥60 ml/min/1.73 m^2^.

**Figure S1.** Mean change in HbA1c to end of treatment according to baseline eGFR∗ (truncated between 5th and 95th percentile) in SUSTAIN 4–6, and 10, and PIONEER 5 and 6.

**Figure S2.** Mean change in HbA1c to end of treatment by eGFR subgroups at baseline treatment group in SUSTAIN 4–6, 10 and PIONEER 5 and 6.

PRISMA 2020 Checklist
