# Supplementary material for: Hemoglobin A1c Reduction With the GLP-1 Receptor Agonist Semaglutide Is Independent of Baseline eGFR: post hoc Analysis of the SUSTAIN and PIONEER Programs
Source: Kidney Int Rep. 2022 Aug 3;7(11):2345–55. doi: 10.1016/j.ekir.2022.07.167 (PMC9751689; doi:10.1016/j.ekir.2022.07.167)
Supplement: Supplementary File (PDF) [file mmc1.pdf]

SUPPLEMENTARY MATERIAL

Table S1. Trial designs

	SUSTAIN 4	SUSTAIN 5	SUSTAIN 6	SUSTAIN 10	PIONEER 5	PIONEER 6
Study drug	OW s.c. semaglutide 0.5 mg / 1.0 mg vs s.c. OD insulin glargine starting from 10 U	OW s.c. semaglutide 0.5 mg / 1.0 mg vs placebo	OW s.c. semaglutide 0.5 mg / 1.0 mg vs placebo	OW s.c. semaglutide 1.0 mg vs s.c. OD liraglutide 1.2 mg	OD oral semaglutide 14 mg vs placebo	OD oral semaglutide 14 mg vs placebo
Trial design	Open-label RCT	Double-blind RCT	Double-blind placebo-controlled CVOT	Open-label RCT	Double-blind RCT	Double-blind placebo-controlled CVOT
Trial duration	30 weeks	30 weeks	104 weeks*	30 weeks	26 weeks	≤83 weeks (event-driven)*
Previous therapy	OAD	Insulin	Drug-naïve / OAD / insulin	OAD	Insulin	–
GLAs	Add-on: MET± SU	Add-on: basal insulin ± MET	Add-on: with or without basal or premixed insulin	Add-on: MET, SU, or an SGLT-2i,	Add-on: basal insulin ± MET or MET ± SU	Standard of care

				alone or in combination		
Kidney function, eGFR (ml/min/1.73 m^2) exclusion criteria	<30	<30	Chronic hemodialysis or chronic peritoneal dialysis	<30	<30 or ≥60	<30

*Median trial duration for SUSTAIN 6 and PIONEER 6. CVOT, cardiovascular outcomes trial; eGFR, estimated glomerular filtration rate; GLA, glucose-lowering agent; MET, metformin; OAD, oral antidiabetes drug; OD, once-daily; OW, once-weekly; RCT, randomized controlled trial; s.c., subcutaneous; SGLT-2i, sodium–glucose co-transporter-2 inhibitor; SU, sulfonylurea.

Table S2. Demographics and baseline characteristics by trial and baseline eGFR in (A) SUSTAIN 4 and 5, (B) SUSTAIN 6 and 10, and (C) PIONEER 5 and 6*

A

**Study**	**SUSTAIN 4**		**SUSTAIN 5**	
**Baseline eGFR (CKD-EPI)**	**<60**	**≥60**	**<60**	**≥60**
***N* (%)**	43 (4.0)	1039 (96.0)	29 (7.3)	367 (92.7)
Age, mean (SD), years	64.3 (9.8)	56.1 (10.3)	67.9 (7.5)	58.1 (9.9)
Female, *n* (%)	17 (39.5)	491 (47.3)	18 (62.1)	156 (42.5)
HbA1c, mean (SD), %	8.2 (1.0)	8.2 (0.9)	8.3 (0.9)	8.4 (0.8)
Body weight, mean (SD), kg	93.0 (20.5)	93.5 (21.9)	89.3 (17.7)	91.9 (21.2)
Diabetes duration, years, median (IQR)	7.5 (5.6–14.5)	7.2 (4.0–11.1)	16.1 (10.1–23.0)	11.9 (7.0–17.0)
Metformin use at baseline, *n* (%)	43 (100)	1039 (100)	22 (75.9)	308 (83.9)
SBP, mmHg, mean (SD)	131.8 (19.2)	132.1 (15.1)	139.4 (17.7)	134.4 (15.8)
DBP, mmHg, mean (SD)	77.0 (8.5)	80.0 (8.5)	80.2 (12.3)	78.9 (9.6)

UACR, mg/g, geometric mean (% covariance)	30.4 (1176)	14.3 (239)	52.2 (765)	21.5 (341)
eGFR (CKD-EPI), mean (SD)	52.7 (6.5)	97.9 (15.7)	50.6 (7.6)	93.4 (15.3)
Kidney impairment, *n* (%)				
None (eGFR ≥90)	0 (0.0)	751 (72.3)	0 (0.0)	229 (62.4)
Mild (eGFR ≥60 to <90)	0 (0.0)	288 (27.7)	0 (0.0)	138 (37.6)
Moderate (eGFR ≥30 to <60)	43 (100)	0 (0.0)	29 (100)	0 (0.0)
Severe (eGFR ≥15 to <30)	0 (0.0)	0 (0.0)	0 (0.0)	0 (0.0)

B

**Study**	**SUSTAIN 6**			**SUSTAIN 10**	
**Baseline eGFR (CKD-EPI)**	**<45**	**45–60**	**≥60**	**<60**	**≥60**
***N* (%)**	399 (12.2)	444 (13.6)	2427 (74.2)	29 (5.0)	548 (95.0)
Age, mean (SD), years	67.6 (8.1)	67.8 (7.4)	63.6 (6.9)	70.4 (6.2)	59.0 (10.1)
Female, *n* (%)	184 (46.1)	177 (39.9)	925 (38.1)	16 (55.2)	234 (42.7)
HbA1c, mean (SD), %	8.6 (1.4)	8.6 (1.4)	8.7 (1.5)	8.1 (0.9)	8.2 (1.0)
Body weight, mean (SD), kg	90.3 (21.9)	94.3 (22.2)	92.0 (20.0)	88.1 (21.6)	97.4 (21.2)
Diabetes duration, years, median (IQR)	16.3 (10.9–22.6)	14.5 (9.6–20.4)	12.1 (7.2–17.5)	13.4 (9.4–17.5)	8.1 (4.6–12.1)
Metformin use at baseline, *n* (%)	117 (29.3)	267 (60.1)	2014 (83.0)	24 (82.8)	523 (95.4)
SBP, mmHg, mean (SD)	138.7 (20.8)	134.6 (17.0)	135.3 (16.4)	138.7 (14.7)	136.3 (14.8)
DBP, mmHg, mean (SD)	76.1 (11.1)	75.6 (10.7)	77.5 (9.6)	74.7 (9.7)	81.6 (9.3)
UACR, mg/g, geometric mean (% covariance)	121.3 (1501)	34.6 (828.4)	17.4 (486.7)	Not available	Not available
eGFR (CKD-EPI), mean (SD)	34.1 (8.2)	52.6 (4.2)	86.8 (13.7)	49.4 (6.9)	94.6 (14.5)

Kidney impairment, *n* (%)					
None (eGFR ≥90)	0 (0.0)	0 (0.0)	1119 (46.1)	0 (0.0)	369 (67.3)
Mild (eGFR ≥60 to <90)	0 (0.0)	0 (0.0)	1308 (53.9)	0 (0.0)	179 (32.7)
Moderate (eGFR ≥30 to <60)	289 (72.4)	444 (100)	0 (0.0)	29 (100)	0 (0.0)
Severe (eGFR ≥15 to <30)	100 (25.1)	0 (0.0)	0 (0.0)	0 (0.0)	0 (0.0)

C

**Study**	**PIONEER 5**			**PIONEER 6**		
**Baseline eGFR (CKD-EPI)**	**<45**	**45–60**	**≥60**	**<45**	**45–60**	**≥60**
***N* (%)**	122 (37.7)	171 (52.8)	31 (9.6)	333 (10.5)	523 (16.5)	2308 (72.9)
Age, mean (SD), years	70.6 (8.5)	70.8 (7.3)	66.6 (8.2)	70.3 (7.1)	68.6 (6.7)	65.0 (6.8)
Female, *n* (%)	67 (54.9)	88 (51.5)	13 (41.9)	125 (37.5)	180 (34.4)	695 (30.1)
HbA1c, mean (SD), %	8.0 (0.8)	8.0 (0.7)	7.9 (0.7)	8.0 (1.5)	8.1 (1.6)	8.2 (1.6)
Body weight, mean (SD), kg	90.5 (17.4)	90.4 (17.6)	94.4 (18.3)	92.2 (21.2)	93.9 (21.6)	90.1 (21.1)
Diabetes duration, years, median (IQR)	14.2 (8.9–19.0)	12.6 (7.6–16.2)	12.6 (6.0–17.0)	17.4 (12.2–23.2)	15.3 (10.2–21.3)	13.3 (8.1–18.5)
Metformin use at baseline, *n* (%)	79 (64.8)	137 (80.1)	26 (83.9)	141 (42.3)	355 (67.9)	1953 (84.6)
SBP, mmHg, mean (SD)	138.2 (16.4)	136.9 (14.7)	138.5 (12.4)	135.5 (17.5)	136.3 (18.2)	135.4 (17.5)
DBP, mmHg, mean (SD)	79.3 (9.3)	76.6 (9.2)	77.1 (6.9)	74.4 (10.2)	75.3 (10.0)	76.4 (10.0)
eGFR (CKD-EPI), mean (SD)	37.8 (4.8)	51.3 (3.9)	65.5 (5.8)	37.4 (5.8)	52.3 (4.4)	84.5 (13.6)
Kidney impairment, *n* (%)						
None (eGFR ≥90)	0 (0.0)	0 (0.0)	0 (0.0)	0 (0.0)	0 (0.0)	919 (39.8)

Mild (eGFR ≥60 to <90)	0 (0.0)	0 (0.0)	31 (100)	0 (0.0)	0 (0.0)	1389 (60.2)
Moderate (eGFR ≥30 to <60)	114 (93.4)	171 (100)	0 (0)	304 (91.3)	523 (100)	0 (0)
Severe (eGFR ≥15 to <30)	8 (6.6)	0 (0)	0 (0)	28 (8.4)	0 (0)	0 (0)

Data are from the full analysis set.

CKD-EPI, Chronic Kidney Disease Epidemiology Collaboration; DBP, diastolic blood pressure; eGFR, estimated glomerular filtration rate (measured in ml/min/1.73 m^2); HbA1c, haemoglobin A1c; SD, standard deviation; SBP, systolic blood pressure; SD, standard deviation; UACR, urinary albumin–creatinine ratio.

*AEs are included for participants with ⩾1 event.

Table S3. Summary of adverse events in (A) SUSTAIN 6 and 10, and (B) PIONEER 5 and 6 by baseline eGFR <45, 45 to <60, and ≥60 ml/min/1.73 m^2

A

**Study**	**SUSTAIN 6**						**SUSTAIN 10**			
**Baseline eGFR (CKD-EPI)**	**<45**		**45 to <60**		**≥60**		**<60**		**≥60**	
	Semaglutide (*n* = 193)	Placebo (*n* = 206)	Semaglutide (*n* = 223)	Placebo (*n* = 221)	Semaglutide (*n* = 1215)	Placebo (*n* = 1212)	Semaglutide (*n* = 14)	Liraglutide (*n* = 15)	Semaglutide (*n* = 276)	Liraglutide (*n* = 272)
AEs	176 (91.2)	192 (93.2)	204 (91.5)	202 (91.4)	1076 (88.6)	1081 (89.2)	13 (92.9)	13 (86.7)	192 (69.6)	177 (65.1)
Serious AEs	80 (41.5)	97 (47.1)	102 (45.7)	97 (43.9)	378 (31.1)	429 (35.4)	0 (0)	1 (6.7)	18 (6.5)	22 (8.1)
Severe AEs	58 (30.1)	79 (38.3)	80 (35.9)	69 (31.2)	265 (21.8)	258 (21.3)	2 (14.3)	2 (13.3)	16 (5.8)	15 (5.5)
Fatal AEs	10 (5.2)	12 (5.8)	13 (5.8)	10 (4.5)	39 (3.2)	38 (3.1)	0 (0)	0 (0)	0 (0)	0 (0)
GI AEs	105 (54.4)	79 (38.3)	124 (55.6)	86 (38.9)	612 (50.4)	414 (34.2)	7 (50.0)	5 (33.3)	120 (43.5)	105 (38.6)
Severe hypoglycemic episodes (ADA)	6 (3.1)	10 (4.9)	5 (2.2)	8 (3.6)	14 (1.2)	11 (0.9)	0 (0)	0 (0)	0 (0)	0 (0)
Acute kidney failure	13 (6.7)	26 (12.6)	26 (11.7)	13 (5.9)	25 (2.1)	28 (2.3)	0 (0)	1 (6.7)	0 (0)	0 (0)

AE leading to premature treatment discontinuation	33 (17.1)	23 (11.2)	38 (17.0)	14 (6.3)	143 (11.8)	73 (6.0)	4 (28.6)	1 (6.7)	29 (10.5)	19 (7.0)

B

**Study**	**PIONEER 5**						**PIONEER 6**					
**Baseline eGFR (CKD-EPI)**	**<45**		**45 to <60**		**≥60**		**<45**		**45 to <60**		**≥60**	
	Sema glutide (*n* = 65)	Placebo (*n* = 57)	Sema-glutide (*n* = 83)	Placebo (*n* = 88)	Sema-glutide (*n* = 15)	Placebo (*n* = 16)	Sema-glutide (*n* = 167)	Placebo (*n* = 166)	Sema-glutide (*n* = 267)	Placebo (*n* = 256)	Sema-glutide (*n* = 1150)	Placebo (*n* = 1158)
AEs	45 (69.2)	42 (73.7)	68 (81.9)	56 (63.6)	9 (60.0)	11 (68.8)	79 (47.3)	65 (39.2)	123 (46.1)	93 (36.3)	453 (39.4)	370 (32.0)
Serious AEs	8 (12.3)	10 (17.5)	11 (13.3)	6 (6.8)	1 (6.7)	2 (12.5)	50 (29.9)	53 (31.9)	67 (25.1)	67 (26.2)	207 (18.0)	249 (21.5)
Severe AEs	3 (4.6)	7 (12.3)	6 (7.2)	6 (6.8)	1 (6.7)	2 (12.5)	33 (19.8)	37 (22.3)	48 (18.0)	49 (19.1)	147 (12.8)	130 (11.2)
Fatal AEs	0 (0)	0 (0)	0 (0)	1 (1.1)	1 (6.7)	1 (6.3)	8 (4.8)	12 (7.2)	4 (1.5)	11 (4.3)	13 (1.1)	23 (2.0)
GI AEs	25 (38.5)	9 (15.8)	44 (53.0)	15 (17.0)	5 (33.3)	4 (25.0)	36 (21.6)	10 (6.0)	45 (16.9)	11 (4.3)	186 (16.2)	52 (4.5)
Severe hypoglycemic episodes (ADA)	0 (0)	0 (0)	0 (0)	0 (0)	0 (0)	0 (0)	2 (1.2)	2 (1.2)	11 (4.1)	1 (0.4)	13 (1.1)	13 (1.1)
Acute kidney failure	1 (1.5)	2 (3.5)	3 (3.6)	1 (1.1)	0 (0)	0 (0)	11 (6.6)	8 (4.8)	2 (0.7)	4 (1.6)	10 (0.9)	10 (0.9)

AE leading to premature treatment discontinuation	8 (12.3)	4 (7.0)	14 (16.9)	4 (4.5)	2 (13.3)	0 (0)	57 (34.1)	37 (22.3)	82 (30.7)	52 (20.3)	285 (24.8)	178 (15.4)

Data are *n* (%) and are from the full analysis set.

ADA, American Diabetes Association; AE, adverse event; CKD-EPI, Chronic Kidney Disease Epidemiology Collaboration; eGFR, estimated glomerular filtration rate (measured in ml/min/1.73 m^2); GI, gastrointestinal.

Figure S1. Mean change in HbA_{1c} to end of treatment according to baseline eGFR* (truncated between 5th and 95th percentile) in SUSTAIN 4–6 and 10, and PIONEER 5 and 6.

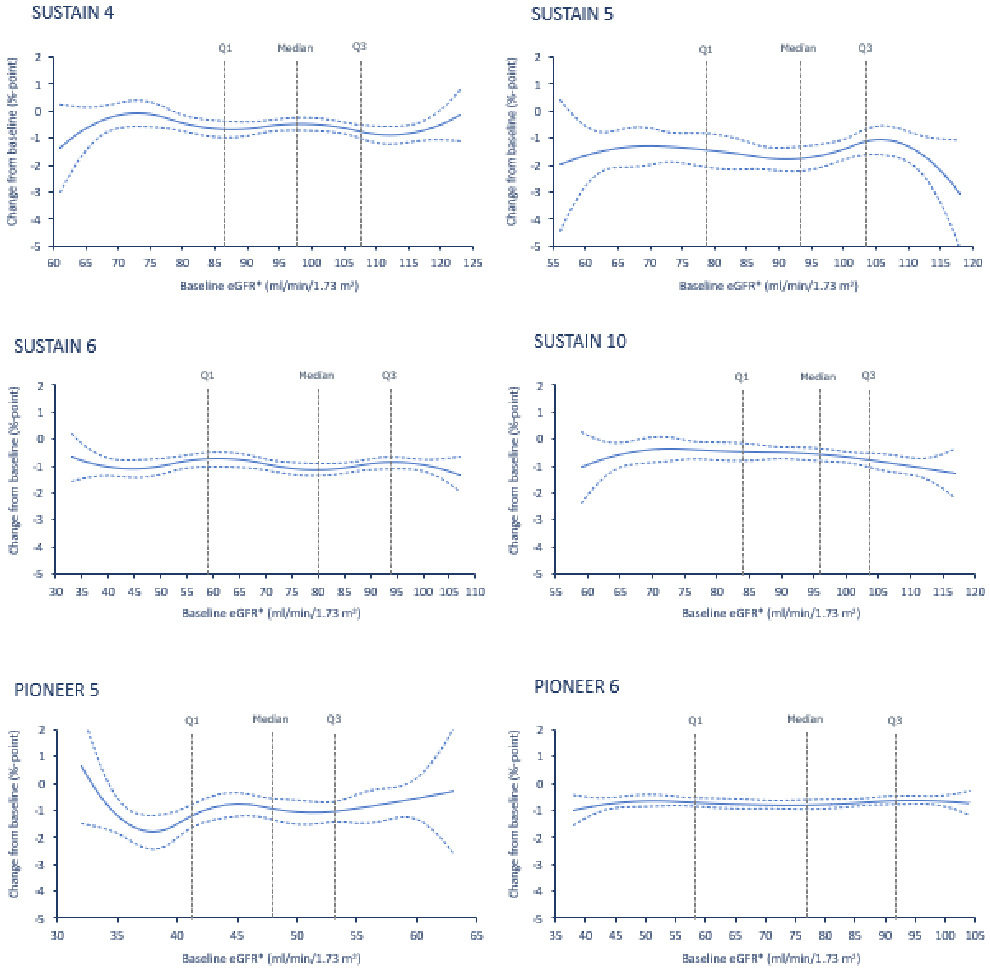

*According to the Chronic Kidney Disease Epidemiology Collaboration equation.

Data are from the full analysis set. Data from participants who were on randomized treatment and without rescue medication or prematurely discontinued were included in the analyses, except for SUSTAIN 6 and PIONEER 6, for which all in-trial data for randomized participants were included. The treatment difference with baseline eGFR as a continuous variable was predicted using a mixed model with repeated measures quadratic spline function. Data are truncated between 5th and 95th percentile. Dashed lines represent 95% confidence intervals.

eGFR, estimated glomerular filtration rate.

Figure S2. Mean change in HbA_{1c} to end of treatment by eGFR subgroups at baseline treatment group in SUSTAIN 4–6, 10 and PIONEER 5 and 6.

Trial arms	eGFR*	Participants (n/N)	Mean baseline eGFR (SE)	Mean baseline HbA_{1c} (SE)	Mean [95% CI] change in HbA_{1c} from baseline to EOT	Mean change in HbA_{1c} from baseline to EOT, %-point	95% CI	p-value
**SUSTAIN 4**								
Semaglutide	<60	22/29	51.8 (7.2)	8.3 (1.0)		−1.7	[−2.1;−1.3]	0.15
	≥60	549/693	97.4 (16.0)	8.2 (0.9)		−1.4	[−1.5;−1.3]	
Insulin glargine	<60	13/14	54.6 (4.3)	7.9 (0.9)		−1.0	[−1.5;−0.5]	0.41
	≥60	309/346	98.8 (15.0)	8.1 (0.9)		−0.9	[−0.9;−0.7]	
**SUSTAIN 5**								
Semaglutide	<60	18/20	50.7 (8.1)	8.2 (0.9)		−1.6	[−2.0;−1.2]	0.94
	≥60	201/243	93.7 (14.7)	8.3 (0.8)		−1.6	[−1.8;−1.5]	
Placebo	<60	5/9	50.4 (7.0)	8.4 (1.1)		−0.5	[−1.2;0.3]	0.24
	≥60	89/124	92.8 (16.6)	8.4 (0.9)		−0.1	[−0.3;0.1]	
**SUSTAIN 6**								
Semaglutide	<45	137/193	34.5 (8.1)	8.7 (1.4)		−1.4	[−1.6;−1.2]	0.49
	45 to <60	155/223	52.7 (4.4)	8.6 (1.4)		−1.4	[−1.6;−1.2]	
	≥60	900/1,215	86.3 (13.7)	8.7 (1.5)		−1.4	[−1.5;−1.3]	
Placebo	<45	133/206	33.8 (8.3)	8.5 (1.4)		−0.4	[−0.6;−0.2]	0.44
	45 to <60	159/221	52.4 (4.0)	8.6 (1.4)		−0.5	[−0.7;−0.3]	
	≥60	943/1,212	87.2 (13.7)	8.8 (1.5)		−0.4	[−0.5;−0.3]	
**SUSTAIN 10**								
Semaglutide	<60	8/14	51.5 (5.9)	7.8 (0.8)		−1.7	[−2.1;−1.2]	0.95
	≥60	233/276	95.0 (13.9)	8.2 (0.9)		−1.7	[−1.8;−1.6]	
Liraglutide	<60	13/15	47.3 (7.4)	8.4 (0.9)		−0.9	[−1.3;−0.5]	0.78
	≥60	231/272	94.3 (15.1)	8.3 (1.0)		−1.0	[−1.1;−0.9]	
**PIONEER 5**								
Oral semaglutide	<45	54/65	37.4 (5.0)	8.0 (0.7)		−1.2	[−1.5;−1.0]	0.71
	45 to <60	60/83	51.2 (3.9)	8.0 (0.7)		−1.1	[−1.3;−0.9]	
	≥60	12/15	64.5 (5.4)	7.9 (0.6)		−1.1	[−1.5;−0.6]	
Placebo	<45	43/57	38.2 (4.7)	7.9 (0.8)		0.0	[−0.3;0.2]	0.64
	45 to <60	71/88	51.5 (3.9)	8.0 (0.7)		−0.2	[−0.4;0.0]	
	≥60	13/16	66.4 (6.1)	7.9 (0.9)		−0.1	[−0.5;0.4]	
**PIONEER 6**								
Oral semaglutide	<45	121/167	37.0 (5.9)	8.0 (1.4)		−1.1	[−1.3;−0.9]	0.28
	45 to <60	217/267	52.5 (4.3)	8.2 (1.5)		−1.0	[−1.2;−0.9]	
	≥60	952/1,150	84.6 (13.6)	8.2 (1.6)		−1.1	[−1.1;−1.0]	
Placebo	<45	132/166	37.9 (5.8)	8.0 (1.5)		−0.2	[−0.4;−0.1]	0.16
	45 to <60	210/256	52.0 (4.4)	8.0 (1.7)		−0.4	[−0.5;−0.3]	
	≥60	1,001/1,158	84.4 (13.6)	8.2 (1.6)		−0.3	[−0.4;−0.3]	

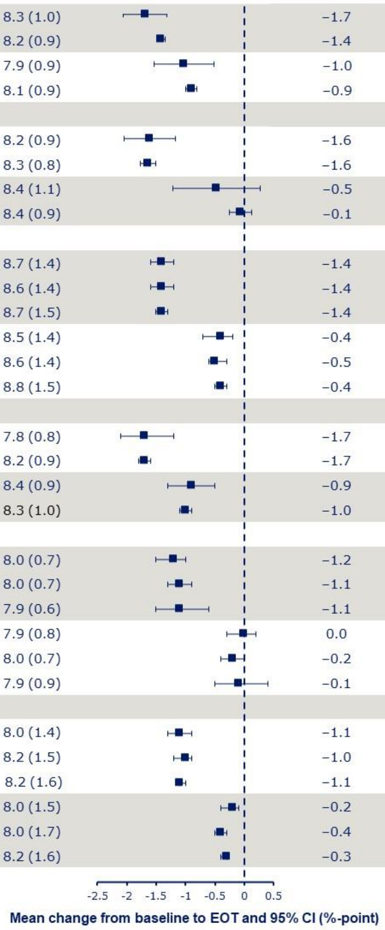

*eGFR is measured in ml/min/1.73 m^2.

Data are from the full analysis set. Data from participants who were on randomized treatment and without rescue medication or prematurely discontinued were included in the analyses, except for SUSTAIN 6 and PIONEER 6, for which all in-trial data for randomized participants were included.

CI, confidence interval; eGFR, estimated glomerular filtration rate; EOT, end of treatment; N, number of participants in the full analysis set who received

semaglutide; n, number of participants who received semaglutide and contributed to the analysis; SE, standard error.
